# Epidemiology, Clinical Characteristics, and Management Status of Hepatitis B: A Cross-sectional Study in a Tertiary Care Hospital at Karachi, Pakistan

**DOI:** 10.7759/cureus.3880

**Published:** 2019-01-14

**Authors:** Nazish Butt, M Ali Khan, Farhan Haleem, Sehrish Butt, Sehrish Reema, Talha Qureshi, Amanullah Abbasi

**Affiliations:** 1 Gastroenterology, Jinnah Postgraduate Medical Centre, Karachi, PAK; 2 Internal Medicine, Jinnah Postgraduate Medical Center, Karachi, PAK; 3 Biomedical and Biological Sciences, Dow University of Health Sciences, Karachi, PAK; 4 Internal Medicine, Jinnah Postgraduate Medical Centre, Karachi, PAK; 5 Internal Medicine, Dow University of Health Sciences, Karachi, PAK

**Keywords:** hepatitis b virus, epidemiology, socio-demographic and clinical characteristics, treatment

## Abstract

Background

Hepatitis B virus (HBV) infection is a serious health problem in Pakistan. In view of the serious socioeconomic consequences, identifying patient characteristics and the current treatment for the disease will enhance HBV regulation and its medical management.

Aims

To describe the epidemiology, clinical characteristics, and current management status of patients infected by HBV.

Methods

We undertook an observational, cross-sectional, and epidemiological study at the Jinnah Postgraduate Medical Centre, Karachi, during the period from January 2014 to November 2017. Male and female patients of any age and with documentation for an HBV infection were eligible for inclusion in the study. An HBV infection was defined as a positive hepatitis B surface antigen test.

Results

A total of 500 patients were analyzed. The mean age at presentation was 29.86±13.68 years. The majority of the patients (25.6%) were ethnically Sindhi followed by Pathan (24.4%), indicating a high prevalence among the rural-based population of Pakistan. The mean duration of the disease was 3.51±4.46 years. The most common cause for the spread was positive family history (40.4%) followed by roadside barbers (30.0%). Most patients were Child-Pugh (CP) class A (84.6%) and the median Modified End-Stage Liver Disease (MELD) score was 7. Upper gastrointestinal bleeding was the most frequent hepatic complication (6.2%). Antiviral medications had been received by 18.6% of patients previously. Peg-interferon (6.0%) was the major antiviral medication prescribed to treatment-experienced patients.

Conclusions

This observational, real-life study has identified some gaps between clinical practice and guideline recommendations in Pakistan. To achieve better health outcomes, several improvements, such as disease monitoring and optimizing antiviral regimens, should be made to improve disease management.

## Introduction

Hepatitis B virus (HBV), a blood-borne virus, is the leading cause of chronic liver disease (CLD) in the developing world and remains a major cause of the morbidity and mortality associated with CLD [1].

Pakistan is in the intermediate endemicity region, with an estimated carrier rate of 3%-5% [2]. HBV remains a debilitating disease, especially for the province of Sindh [3], with some estimates putting the disease burden as high as 8 million patients in the province. This is especially significant in the setting of tertiary care, where chronic liver disease is presenting with an ever-increasing burden and HBV is one of the leading causes.

Even with such a high burden of HBV, few studies have been done to evaluate the trends and characteristics of HBV presentation at the tertiary care level, as most patients are lost to follow-up [4].

## Materials and methods

This cross-sectional study was carried out at Jinnah Postgraduate Medical Centre, the largest tertiary care hospital in Karachi, Pakistan, between January 2014 and September 2017, at Medical Unit IV, Gastroenterology and Hepatology section. A total of 500 patients were recruited based on a positive result for hepatitis B surface antigen (HBsAg), indicating a chronic hepatitis B (CHB) infection, irrespective of the disease activity, duration, and infectivity.

Measurement of epidemiological and clinical characteristics

In the present study, the basic epidemiological data of the patients, including age, gender, occupation, risk factors, and ethnicity, were recorded. Moreover, the clini­cal information of patients such as disease manifestations and complications were also collected in the present study.

Laboratory tests and treatment regimen

All 500 patients were hepatitis B surface antigen (HBsAg) positive and were followed up with a complete blood count (CBC), liver function tests (LFTs), prothrombin time (PT), international normalized ratio (INR), urea and creatinine levels, ultrasound abdomen, hepatitis B envelope antigen (HBeAg), and antibody (HBeAb), and HBV deoxyribonucleic acid (DNA) polymerase chain reaction (PCR) quantitative and anti-delta antibody (ADV Ab) titers. Quantitative PCR for hepatitis delta virus (HDV) was carried out in all ADV Ab-positive patients. Serology for co-infection with HCV was also sent in all cases.

In selected cases, computed tomography (CT) was performed with alpha-fetoprotein levels (AFP) for the detection of hepatocellular carcinoma (HCC). When suspected, workup for concomitant diseases such as Wilson’s disease was also performed.

Patients had to give a complete clinical record of any previous medications taken for HBV, if at all. Based on all parameters, the disease was classified and managed according to the latest guidelines.

Statistical analysis

Data were analyzed using the Statistical Package for the Social Sciences (SPSS) version 21.0 software (SPSS Inc., Chicago, IL, USA). The data were presented as mean ± standard deviation (SD). The comparisons were performed with a paired t-test, and p<0.05 was considered statistically significant.

## Results

Baseline and demographic characteristics

Patients were predominantly male (74.8%), with most being residents of Karachi (68.6%). The mean age of the patients was 29.86±13.68 years and the duration of disease since diagnosis was 3.51±4.46 years. Most patients (85%) had Child-Pugh class A disease. Baseline characteristics are shown in Table 1. Demographics are shown in Table 2.

**Table 1:** Baseline characteristics HBV: Hepatitis B virus

	Mean
Age	29.86±13.68 years
Duration of HBV	3.51±4.46 years
Hemoglobin	12.81±2.48 g/dl
Mean Corpuscular Volume	79.76±16.90 fl
Total Leukocyte Count	07.48±5.74 x10^9^/L
Platelets	220.48 ±92.47 x10^9^/L
Prothrombin Time	11.25±3.75 seconds
International Normalized Ratio (INR)	1.04±0.642
Alanine Aminotransferase	72.63±214.47 U/L
Albumin	3.68±1.12 g/dl
Creatinine	0.811±0.282 mg/dl
Total Bilirubin	1.156±2.258 mg/dl
Alkaline Phosphatase	225.20±190.01 U/L

**Table 2:** Demographics

	N=500(%)
Gender	
Male	374(74.8%)
Female	126(25.2%)
Marital Status	
Married	283(56.6%)
Unmarried	217(43.4%)
Residence	
Karachi	343(68.6%)
Non-Karachi	126(25.2%)

Being a busy metropolitan, we received patients from all walks of life and ethnicities. Students (34.4%) and housewives (18.8%) were most commonly affected by CHB. Ethnically, Sindhis (25.6%) had the highest prevalence of CHB followed by Pathans (24.4%). The occupations and ethnicity of the patients are shown in Table 3 and Table 4, respectively.

**Table 3:** Occupation

Occupation	N(%)
Student	172(34.4%)
Housewife	94(18.8%)
Laborer	62(12.4%)
Teacher/Imam	38(7.6%)
Unemployed	32(6.4%)
Farmer	24(4.8%)
Shopkeeper	22(4.4%)
Odd jobs	20(4.0%)
Office worker	12(2.4%)
Forces	12(2.4%)
Driver	7(1.2%)
Businessman	3(0.6%)
Cook	2(0.4%)

**Table 4:** Ethnicity

Ethnicity	N=500 (%)
Sindhi	128(25.6%)
Pathan	122(24.4%)
Balochi	102(20.4%)
Urdu Speaking	55(11.0%)
Punjabi	47(9.4%)
Others	46(9.2%)

Age distribution

Based on the data collected, the patients were divided into six age groups, ≥18 years, 19-29 years, 30-39 years, 40-49 years, 50-59 years, and ≥60 years. The highest numbers of HBV-infected patients were in the 20-29 age group, making up 32.6% of all patients. This was followed by the ≤18 age group, making up 24.6% of all patients. There was a male preponderance for all age groups. Age distribution (for male and female) is shown in Table 5.

**Table 5:** Age distribution

Age groups (years)	Male(N)	Female(N)	Overall(%)
≤18	109	14	24.6%
19-29	120	43	32.6%
30-39	68	32	20.0%
40-49	44	19	12.6%
50-59	15	11	5.20%
≥60	18	07	5.0%

Risk factors

Multiple risk factors were identified for the transmission of HBV. A positive family history of previous HBV infection was the predominant risk factor; it was present in 40.4% of our patients. Males had more exposure to all of the risk factors, as summarized in Table 6, with the comparative analysis seen in Table 7.

**Table 6:** Risk factors HBV: Hepatitis B virus

	N=500(%)
HBV +ve Family member	202(40.4%)
Roadside barber	149(29.8%)
Multiple injections	147(29.4%)
Dental extraction	90(18.0%)
Blood transfusion	73(14.6%)
Surgery	69(13.8%)
Multiple sex partners	29(5.8%)
Tattoos	14(2.8%)
I/V drug abuse	6(1.2%)

**Table 7:** Comparative analysis of risk factors for each gender

Risk factors	Male(N)	Female(N)	p-value
Roadside Barber	148	0	.000
Multiple Injections	107	40	>0.005
Blood Transfusion	33	40	.000
Tattoo	9	5	>0.005
Surgery	40	29	.001
Dental Extraction	66	24	>0.005
I/V Drug Abuser	5	1	>0.005
Multiple Sex Partners	29	0	0.001

Clinical characteristics and symptomology

Many patients were asymptomatic or incidentally (56.6%) found out to be HBsAg positive (Table 8). Of those with clinical symptoms, body ache (52.6%) was the most prevalent (Table 9). There was a male preponderance for both groups.

**Table 8:** HBV diagnosis HBV: Hepatitis B virus

N=500	Male (%)	Female (%)
Symptomatic	153(30.6%)	64(12.8%)
Asymptomatic/Incidental	222(44.4%)	61(12.2%)

**Table 9:** HBV signs and symptoms HBV: Hepatitis B virus

	N=500
Bodyache	263(52.6%)
Lethargy	214(42.8%)
Upper abdominal pain	148(29.6%)
Dyspepsia	121(24.2%)
Weight loss	99(19.8%)
Jaundice	91(18.2%)
Melena	39(7.8%)
Ascites	38(7.6%)
Hematemesis	34(6.8%)
Portosystemic Encephalopathy	17(3.4%)
Rash	15(3.0%)

Virological and serological characteristics

Virological characteristics are shown in Table 10. HBV carriers (44.8%) constituted the largest group, followed by HBeAg +ve chronic hepatitis B (16.0%). The prevalence of chronic hepatitis delta (CHD) patients taking part in this study was 11.4%. Three (0.6%) cases of acute hepatitis were observed as well.

**Table 10:** Virological status HBV: Hepatitis B virus; HBeAg: Hepatitis B envelope antigen; HDV: Hepatitis delta virus; HCV: Hepatitis C virus

	N=500(%)
HBV Carrier	224(44.8%)
HBeAg +ve Chronic Hepatitis B	80(16.00%)
HBeAg -ve Chronic Hepatitis B	60(12.00%)
HDV Co-Infection	57(11.4%)
Decompensated Liver Disease	50(10.00%)
Miscellaneous	19(3.80%)
Hepatoma	05(1.0%)
HBV/HDV + HCV Co-Infection	50(1.0%)

Treatment status

Eighty-nine (17.8%) patients had previously received medication for CHB, with Peg-interferon being the most commonly prescribed drug. Despite such a high number of asymptomatic patients and carriers, we initiated a new regimen in 137 (27.4%) patients. The most common drug prescribed was Entecavir. The treatment statuses and treatment offered are summarized in Table 11 and Table 12, respectively.

**Table 11:** Treatment status

	N=500(%)
Treatment Naive	412(82.4%)
Treatment Experienced	88(17.6%
Peg-Interferon (INF)	30(6.0%)
Entecavir	23(4.6%)
Tenofovir	19(3.8%)
Conventional-INF	9(1.8%)
Lamivudine	5(1.0%)
Combination	2(0.4%)

**Table 12:** Treatment offered

	N=500(%)
None	363(72.6%)
Entecavir	91(18.2%)
Peg-INF	25(5.0%)
Tenofovir	21(4.2%)

## Discussion

The mean age at presentation was highly reflective of Pakistan’s status as an intermediate endemicity region, where most acquire the disease during adolescence [5-6], although it also showed that HBV is now affecting a much younger population [7].

The most troubling aspect was the time since diagnosis; patients were aware of having CHB for years and still did not seek any expert help. This can be attributed to a lack of knowledge of the natural course of HBV, vaccination protocols, complications, and implications [8-9].

The baseline characteristics, for the most part, were within normal ranges; this correlates with the asymptomatic nature of CHB and the high number of carriers in our study. High alanine aminotransferase (ALT) levels were observed in acute hepatitis B, active CHB, and co-infection with HDV. All patients with CHD had active disease and disease markers were raised as well.

The demographic data shows that every ethnicity is affected by HBV, and it can no longer be thought of as a disease restricted to one province, a particular area or a race [10-12]; this is also representative of Karachi as a diverse metropolitan for all cultures. However, the representation of residence from within the city or otherwise was arbitrary; most of our patients move in and out of the city for a multitude of reasons.

The high male preponderance for HBV is in part due to men being exposed to more risk factors and probably having better access to health care facilities. There was no significant difference between the marital statuses of patients.

Three-hundred eighty-six (77.2%) of our patients were aged 40 years or below with the age bracket of 18-29 years having the highest prevalence. This is consistent with Khan et al. [13], but we did show a slightly higher prevalence in the 18 years or under group. We have also clearly established a correlation between the age groups and various occupations. The younger patients tended to be students or housewives.

Older patients tended to be laborers or teachers. Although no occupation conferred a higher risk for HBV transmission, the proportion of students and teachers is too large to ignore and calls for mass screening protocols and subsequent management at all levels of institutionalized education, be it regular schools, colleges, universities or madrasas.

The risk factors for the transmission of HBV have been clearly identified in our study. A high association with chronic HBV was found with dental procedures, roadside barbers, and positive family history for HBV. Unfortunately, the association with all risk factors has only increased or remained the same when compared with previous studies [14].

The route of transmission could not be always be ascertained. Some patients didn’t have exposure to any of the risk factors or, at least, didn’t know about it. Despite a large number of patients being related to a diagnosed case of HBV, whether it be a parent, sibling, cousin, or relative. The disease status in that particular relative was largely unknown; only the status of HBsAg being positive was known for most cases.

Family members already having CHB understate another problem: that there is a subset of our population, which in all likelihood has CHB and is not aware of it or is not seeking medical advice with regards to it. Furthermore, they confer a great risk of transmitting HBV to new subjects. Immediate workup and possible interventions are needed, as this represents the most amenable risk factor in our study for the time being.

Two-hundred seventeen (43.4%) patients had some symptom, for which an HBsAg test was carried out. Body aches and lethargy were the predominant symptoms, but it was not possible to contribute these symptoms purely to CHB, as most patients with the afore-mentioned symptoms were either carriers or were incidentally found out with no stigmata of chronic or active disease. With the exception of 50 (10.0%) cases of decompensated liver disease (DCLD), symptoms could not be matched with disease activity.

Two-hundred eighty-three (56.6%) patients were asymptomatic or incidentally found out during screening. The majority of these were either found out as part of clinical fitness tests for work visas or during blood donation; more patients were diagnosed during blood donation. As such, the seroprevalence of HBV for blood donors remains a major problem for the country [15-17].

While many studies have looked at the serological status of CHB in Pakistan [18], our study classifies various presentations of disease patterns, taking into account viral markers, infectivity status, laboratory data, and symptomology. Therefore, it is unique for Pakistan to the best of our knowledge.

Most (44.8%) of our patients were inactive HBV carriers; they were advised to be vigilant with follow-up every six months due to the risk of developing cirrhosis and HCC despite the carrier state [19]. Eighty (16.0%) patients had HBeAg positive (+ve) CHB and 60 (12.0%) had HBeAg negative (-ve) CHB.

Pakistan has one of the highest prevalence of chronic HDV (CHD) worldwide, with some areas reporting it to be as high as 35% to 88% [20]. The prevalence of chronic HDV was 11.4% in our study, much lower than previously reported [21-22]. This can be attributed to geographical variation. The disease pattern was uniform, with almost all having active disease, increased ALT levels, and other disease markers that were consistent with earlier studies. It was also associated with higher rates of DCLD.

All patients who were previously diagnosed as CHD had been given Peg-interferon, making it the most prescribed drug (6.0%) in the treatment-experienced group. As reported previously, the response rate to Peg-interferon was dismal [23]; only one patient had undetectable HDV ribonucleic acid (RNA) levels in the treatment-experienced group. All patients who did not respond to Peg-interferon for HDV were shifted to tenofovir.

The decision to start a new drug or modify a previous regimen was a multifactorial one. We only prescribed Peg-interferon to our non-decompensated cases of CHD. For those who did not respond to previous treatment or had developed DCLD, we switched to Tenofovir, as mentioned earlier. All patients that had developed DCLD were required to go through further testing, such as screening endoscopies and/or CT scans with the hepatoma protocol. The definitive need for liver transplantation in a DCLD patient was all too apparent, but such a facility is beyond the reach of the majority of our population.

Entecavir was our drug of choice for patients of CHB that had active disease, positive infectivity status, and/or active replication. The choice was primarily based on easier availability and cost-effectiveness. It was prescribed to 91 (18.2%) patients. Most patients (72.6%) did not require any treatment at all.

## Conclusions

HBV remains a conundrum. For the most part, it runs a silent course without symptoms or manifestations. This very fact makes it problematic and difficult to diagnose; its silent nature masks its deadly potential. HBV is now affecting an ever-younger population and, more importantly, it’s highly prevalent in our educational institutes.

Risk factors for HBV transmission have not changed over the last two decades. The same can be said about demographics; HBV now effects most occupations across all ethnicities. Oral anti-viral medication remains the drug of choice of CHB; they are better tolerated with far fewer side effects and are more cost-effective. There is no effective treatment of co-infection with HDV and progression to cirrhosis and decompensation is academic.

Improved patient education, better vaccination protocols/programs, easier access to qualified specialists, reasonably priced medication, open communication with discussions, improved sanitation and hygiene, and proper sterilization of not just surgical but everyday instruments (razor, clippers, etc.) are just a few areas where new standards and protocols need to be set to tackle this endemic.
